# The EU Strategy on the Rights of the Child and the European Child Guarantee—Evidence-Based Recommendations for Alternative Care

**DOI:** 10.3390/children8121181

**Published:** 2021-12-14

**Authors:** Bárbara Mourão Sacur, Elisete Diogo

**Affiliations:** 1 Católica Research Centre for Psychological, Family and Social Wellbeing (CRC-W), Universidade Católica Portuguesa, 1649-023 Lisboa, Portugal; barbaramourao@hotmail.com; 2 Centro Interdisciplinar de Ciências Sociais (CICS.NOVA), Faculdade de Ciências Sociais e Humanas, Universidade NOVA de Lisboa, 1069-061 Lisboa, Portugal; 3 Research Centre for Endogenous Resource Valorization (VALORIZA), Polytechnic Institute of Portalegre (IPP), 7300-110 Portalegre, Portugal

**Keywords:** EU Strategy on the Rights of the Child, European Child Guarantee, alternative care, child protection system, de-institutionalisation, foster care, kinship care, special guardianship, Portugal

## Abstract

Protection and promotion of child rights are referred to as a central purpose of the European Union (EU). Therefore in 2021, the EU Strategy on the Rights of the Child and the European Child Guarantee were published to enable children to have the best possible life in the EU and worldwide. Member states were invited to implement the directions of both documents into practice. The present study analyses and showcases the evidence on how to progress implementation of the Strategy and the Guarantee regarding alternative care in Portugal. A literature review was conducted based on international literature. Evidence-based recommendations for the Portuguese transition process towards quality, family and community-based care are stated. De-institutionalisation and strengthening specific services—kinship care, special guardianship, and foster care—are advocated, namely specialising the workforce, and promoting training for kinship carers and prospective special guardians. To conclude, the revision and monitoring of the measures for children in need of alternative care are suggested as well as integrating and publishing data from the diverse services of the alternative care system.

## 1. Introduction

Protection and promotion of the child’s rights are denoted as fundamental purposes of the European Union (EU). Therefore in 2021, two relevant documents—the EU Strategy on the Rights of the Child (abbreviated form in the present paper: “the Strategy”) and the European Child Guarantee (abbreviated form: “the Guarantee”)—are proclaimed for assuring the best possible life for children.

The Strategy pursues the shared responsibility to fulfil the rights of every child and to build together with children equal societies for all [1]. In a constant changing world, children can face several challenges, such as violence, discrimination, online abuse, among others, as is highlighted by the Strategy. The COVID-19 pandemic has intensified challenges and inequalities. For example, distance learning affected children lacking internet, those with special needs and very young students [1]. The Strategy addresses several challenges and proposes concrete action.

The Guarantee complements the Strategy, targeting children in need who are under the age of 18 years and who are at risk of poverty and social exclusion [2]. EU Member States should identify the needs of specific groups of children, namely, children in alternative (especially institutional) care. Negative stigmas surrounding children in alternative care continue to differentiate them from others [3]. Those who grow up without family support or at risk of being separated from their family are among the most disadvantaged groups in society because their needs may not be taken into account, especially when they are in low quality alternative care [4].

The implementation of international recommendations consists of a complex process. It is argued that implementation of services should be supported by evidence from research [5]. The lack of attention in this matter from academics lead to the relevance and novelty of the present study, which intends to increase the understanding of the topic.

This study aims to analyse and discuss the implementation of the Strategy as well as the Guarantee on alternative care in Portugal. It contributes to enabling social policy and action to eradicate child social exclusion and to support children at risk and their family. Scientific knowledge is explored with a view to providing evidence-based recommendations.

Accordingly, the paper delimits the concepts of alternative care for clarity, prior to describing the Portuguese context and the key components of the Strategy and the Guarantee. A literature review is presented with robust data in order to support arguments and discussion. Although the Strategy and the Guarantee highlight the situation of children who leave institutional care, the topic will not be detailed in this alternative care paper.

### 1.1. Alternative Care: Concepts

Concepts of alternative care may differ around the world, and also the variety of alternative care typologies cannot correspond exactly to generic descriptions [6]. Therefore, the concepts used in this paper are initially presented.

Children are in alternative care when they are deprived of parental care or at risk of being so [6]. In 2009, the United Nations General Assembly developed the Guidelines for the Alternative Care of Children that emphasize in guideline 5: “Where the child’s own family is unable, even with appropriate support, to provide adequate care for the child, or abandons or relinquishes the child, the State is responsible for protecting the rights of the child and ensuring appropriate alternative care, with or through competent local authorities and duly authorized civil society organizations” [7] (p. 3).

With respect to the setting where the alternative care is provided, the Guidelines for the Alternative Care of Children acknowledge in guideline 53, priority to family and community-based care [7]. In family-based care, an existing family is the care provider, such as in: (i) kinship care—within the child’s extended family or another carer close to the family and known to the child; (ii) foster care—within accredited couples or individuals in their own homes; or (iii) other family-based care. Cantwell and colleagues [6] specify that the other family-based care includes settings where a family plays a care role identical to foster care but does not function within the foster care service. Families who act like guardians for children with long-term alternative care needs are examples of other family-based care. In a complementary way, community-based care refers to the idea that the child lives alongside other people and the community is involved in the process of the child’s recovery [8]. The effects of growing up in a family environment on a child’s life are widely recognized [9,10,11,12,13], centred on the possibility of achieving a sense of belonging, a family model, long term relationships and attachment, educational and labour support as well as a social life.

In the referred guidelines, all alternative care typologies with a non-family-based setting are classified as residential. Several concerns are constantly referred to regarding residential care: “The research on children’s development while in residence is consistent in showing that their physical growth as well as mental and socioemotional development and behavior are substantially delayed” [14] (p. 88). It is a fact that carers and shifts are inconsistent, the number of children per carer is large. Therefore, the quality of care is low [14,15], based on professionals’ reduced training and competence [15]. For many advocates and activists, the continued existence of such places constitutes a massive abrogation of the rights of children [16], particularly the large residential facilities (also called institutions), where the individualised needs of the children are not considered and there can be segregation from the outside world [6].

De-institutionalisation is the process of replacing institutional care for children with care in a family or family-like environment in the community [7], not limited to the children leaving institutions. De-institutionalisation policy focuses on two broad areas: (a) developing family support measures to prevent the separation of children from their family; and (b) developing family-based care placements in order to move children out of the institutions, and to provide options for children who will need ‘alternative care’ placements in the future [16,17]. 

The concepts presented here offer a common framework for understanding the Strategy, the Guarantee, and the Portuguese context of alternative care. 

### 1.2. The Portuguese Context 

The Portuguese child protection system was tailored within the creation of minors’ protection commissions [18,19], privileging a social intervention approach wherein child related agencies and services were gathered to tackle the social problems in cooperation with children and their families [20]. 

In the late 1990s, a comprehensive reform of the legislation relating to minors took place, patterning the current Portuguese child protection system. The Law of the Protection of Children in Danger [21] published at that time and still in force with some later adjustments [22,23,24,25], delineates the competent bodies responsible for child protection and the child protection measures, among other themes. Child Protection Commissions are one of the child protection competent non-judicial bodies that inherited the social intervention approach from the disestablished minors’ protection commissions. These non-judicial bodies compose the initial level of intervention with children in need of protection. The courts and public attorneys are the competent bodies responsible for judicial intervention. These judicial bodies intercede mainly when the Child Protection Commissions actions were not successful. Children in need of protection are persons under 18 years old, or under 21 years old when they require maintenance of protection, as well as under 25 years old if in addition to the maintenance request they are taking an educational course.

Regarding child protection measures, the list is defined as follows: (i) “Support the child in parental care”; (ii) “Support the child in care with other family member”; (iii) “Entrust the child to a reliable and familiar person” that does not belong to the child’s family; (iv) “Support to supervised independent living”, for children older than 15 years old; (v) Foster care; (vi) Residential care; and (vii) “Entrust the child for further adoption to prospective adoptive parents, a foster family or residential care” [21]. 

The child protection measures can involve social, psycho-pedagogical, and financial support to children and their families. On the other hand, foster care also involves the approval and training of a family in order to provide alternative care to a child who does not belong to the family.

Special guardianship (*Apadrinhamento civil*) is not considered a child protection measure, but a civil one approved by a court [26,27] (in its current wording). It allows a long-term placement in a family environment when parents are not in position to assume the parental responsibilities properly, and adoption cannot be envisaged. Special guardianship is suitable for children in need of protection (although it is not exclusively) since it reinforces de-institutionalisation as well as affective bonds between child and carer in a family environment [28]. It is dissimilar from foster care, as a result of permanency and non-involvement of financial support to special guardians.

The terminology for child protection and civil measures presented is not framed in the international concepts used in the Guidelines for the Alternative Care of Children [7]. In Table 1, we propose a categorisation of the child protection and civil measures, analysed in this study by matching them with the referred guidelines.

#### 1.2.1. What Are Facts and Figures Saying?

According to the Portuguese law, the measure “support the child in parental care” must prevail in order to keep the child in her/his family. If parental care is not in the best interest of the child, she/he must be in care with another family member or a reliable and familiar person. Only after these possibilities have been exhausted, foster or residential care are considered. Foster care (in Portugal foster carers do not have blood ties) should be privileged over residential care, namely, for children up to 6 years old (some exceptions are acceptable). 

Out of law documents, facts and figures represent a relevant source to describe the practice. We notice that countries do not have a similar data collection system on children in alternative care, and that different concepts and measures are used, which may complicate the conduct and comprehension of comparative studies [29,30]. We consider that Portugal is one of the countries that should reinforce the presentation of statistics in this domain. Therefore, details on the Portuguese situation are stated below considering an accurate search in available databases. Hence, the national numbers are only available on the following measures: residential care; foster care; and special guardianship. Therefore, the information about the other measures is restricted to those numbers of Child Protection Commissions’ practice.

In 2020, Child Protection Commissions took the measure “support the child in parental care” in 81% of the situations where a child was in need of protection [31]. 

**Figure 1:**
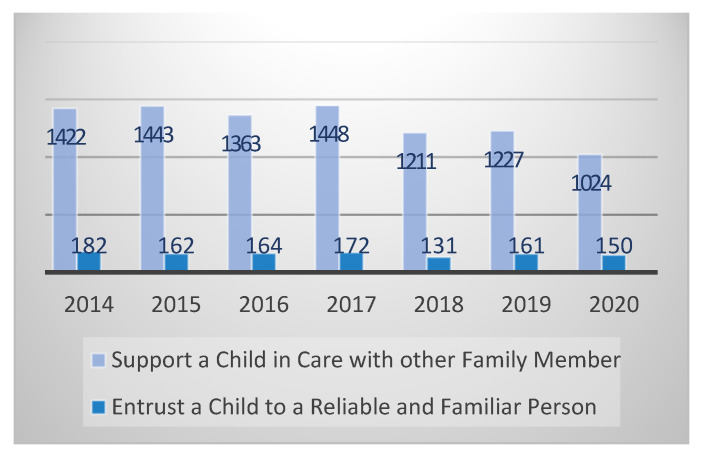
Number of children in kinship care in Portugal (2014–2020). Source: own elaboration based on CNPDPCJ [31].

Current numbers state there are 1,930,689 children living in Portugal [32]. Of these, in relation to kinship care, it is possible to identify 1024 children (8.6%), within ‘support the child in care with other family member’, and 150 children (1.2%) are entrusted to a reliable and familiar person (Figure 1).

Courts present a complementary data collection system; however, certain indicators are not available, namely the typology of the child protection measure. It is possible to observe that in 2020, 3736 children were within a judicial process of child protection in a Family and Minors Court [33], more than the previous year (3429). 

In 2020, in a national perspective, a total of only four special guardianship’s compromises were homologated by a court, significantly less than previously. For example, in 2019 there were 11; in 2018 there were five, and in 2017 there were 11 [33]. In 2020 the Child Protection Commissions proposed the special guardianship for six children in need, of these only two special guardianships were made effective [31].

In the same year, about 6706 children were placed in residential or foster care [34]. Among these, only 202 (3%) children were in a foster family and the others were in residential care (6504 children, corresponding to 97%) [34].

**Figure 2:**
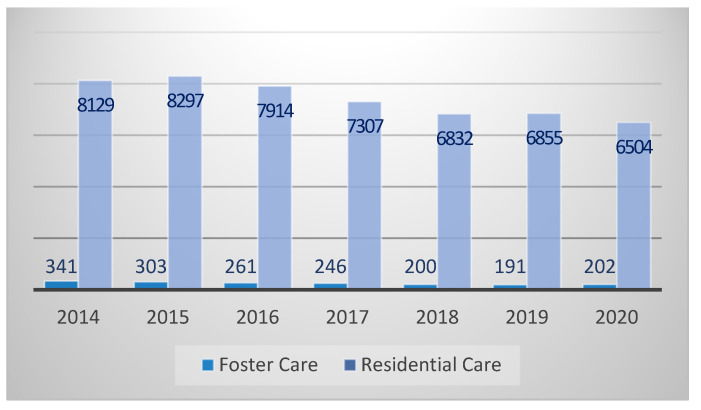
Number of children in alternative care in Portugal (2014–2020). Source: own elaboration based on *CASA Report* [34].

A decreasing trend of children in residential and foster care from 2014 until 2020 is observed in Figure 2, less 1764 children [34]. In 2014, there were 341 children in a foster family and in 2020 there were 202. For children in residential care, in 2014 there were 8129 and in 2020 they were 6504. One possible reason, among others, may be related to a crisis in birth-rate over the years in Portugal [32], consequently the number of children in residential care and in foster care decreased [34]. Therefore, it does not represent an investment in family-based care.

Nevertheless, 2019–2020 registered an increase of 6% in foster care placements, reversing the trend of children in a foster family. This happened within a very large increase of children from 0 to 5 years old, representing 18.4% (38) of all children in a foster family [34], by operation of law [21]. But in this age group, 858 are in residential care [34].

A critical aspect is permanency, foster care and residential care are transitory placements, but the numbers illustrate long-term placements. On average, a child stays in these placements for 3.4 years; in foster care, children stay an average of 6 years (47% longer) [34]. Another critical feature is geographic inequality. The number of foster families available have been more significant in the north of the country and recently in Lisbon [34]. It means that several regions of the country do not have foster families.

After a reinforcement of the legal framework in 2019 and 2020 [35,36] highlighting the importance of growing up in a family context, figures remain low. 

**Figure 3:**
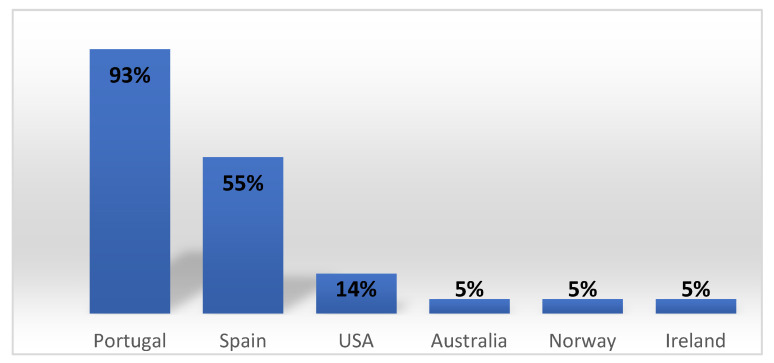
Percentage of children in residential care in different countries. Source: own elaboration based on Furey and Canavan [39] and Ministerio de Derechos Sociales y Agenda 2030 [40].

A different trend is observed around the world. When compared to residential care, foster care is recorded to be the most applied resource within alternative care in Europe and abroad [37], towards a de-institutionalised path [16]. For example, fostered children represent nearly 75%, about 56,500, of all children in care in England [38]. In Ireland, foster care and kinship foster care represent 93%, against 5% in residential care; in Norway, they represent 89% against 5% in residential care; in the United States of America (USA), they represent 75% against 14% in residential care; and in Australia, they represent 88% against 5% in residential care [39]. Even the Spanish child protection system, a bordering country, presents a larger investment than the Portuguese. In 2019, there were 19,320 children, representing 45% in a foster family and 55% in residential care [40]. Therefore, Portugal presents a large percentage of children in residential care when compared to other countries, as stated in Figure 3.

#### 1.2.2. The Portuguese National Strategy on the Rights of the Child 

In December 2020, the Portuguese National Strategy on the Rights of the Child (abbreviated form: “the Portuguese Strategy”) was published [18] focusing on the national priorities on the rights of the child (see Appendix A for more details about the Portuguese Strategy). The Committee on the Rights of the Child—United Nations recommended this publication [41,42].

Regarding children in alternative care, the Portuguese Strategy points out, in its second priority, the importance of children growing up in a suitable family context where parenthood is supported and successful. The importance of developing a global and integrated policy for the family is underlined. De-institutionalisation is encouraged and existing services on residential care must be qualified, making them suitable for a child in need deprived of their family environment. Finally, it is briefly stated that special guardianship should increase for children in need. 

Nevertheless, foster care and residential care are translated only in two strategic aims of the Portuguese Strategy: to reinforce the creation of tangible measures that privilege foster care, and to qualify the residential care system. These goals are supported by the articles 20 and 25 of the Convention [43]. Now, the Portuguese Strategy should be operationalised through action plans, and this study may support them.

## 2. Materials and Methods

The aims of this study are: (i) to characterise and discuss the Strategy and the Guarantee; and (ii) to make evidence-based recommendations for the implementation of both on alternative care in Portugal.

Regarding these objectives, the purpose is to contribute to the debate and support of public policies and practices with scientific evidence, within the paradigm of evidence-based policies. It is argued that action plans and national strategies that operationalise the Strategy and the Guarantee are essential for its effective execution. Hence, this paper intends to contribute to designing these strategic documents. 

Initially, the Strategy and the Guarantee were analysed in view of describing each document and their perspectives regarding alternative care for children. This procedure was essential for achieving the first aim of the study.

A question emerged leading to the second aim: “What is evidence-based outlining about alternative care, to support implementation of the Strategy and the Guarantee in Portugal?”. In order to find relevant articles to answer this question, a literature review [44,45] was performed. The search was conducted in the following databases: Cochrane, PubMed, Psychology and Behavioral Sciences Collection, Academic Search Complete, Scopus, Web of Science, and institutional repositories and libraries, such as the Portuguese Ministry of Labour, Solidarity and Social Security library. Certain keywords have been used: implementing guidelines for children; child rights implementation; children rights strategy; alternative care; de-institutionalisation; safeguarding policies; child protection services; foster care implementation; kinship care practices; and special guardianship.

During the search, the last decade was considered: manuscripts from 2011 to 2021. This option is justified namely by the publication of the Guidelines for the Alternative Care of Children in 2010. Moreover, only blind peer review journals have been selected, and academic accuracy verified (e.g., publication year identified). Quantitative and/or qualitative manuscripts written in Portuguese or English were included, specially focusing on European countries. Other sources were used, such as reports and legal frames, namely periodical reports, recommendations, conventions by national and international organisations as well as the legal framework on the topic and statistical databases.

## 3. The EU Strategy on the Rights of the Child and the European Child Guarantee

The Strategy [1], launched in March of 2021, puts the children and the fulfillment of their needs at the core of the EU policies. This Strategy commits the EU to ensuring the best life for children in the EU, but also worldwide. In order to achieve this demanding goal, it delineates specific actions both to Member States and to European Commission.

The Strategy was built on previous international conventions and standards. The United Nations Convention on the Rights of the Child [43] and its three Optional Protocols [46,47,48] were considered, as well as the United Nations Convention on the Rights of Persons with Disabilities [49], and the Council of Europe’s Strategy for the Rights of the Child (2016–2021) [50]. In addition, previous European Commission communications on the rights of the child were integrated. In preparing the Strategy there were substantive contributions from the European Parliament, Member States, child rights’ organisations, an open consultation, and the suggestions of over 10,000 children [1].

The result was a main document with 23 pages and two Annexes. The main document delineates six thematic areas. Each area is one priority for EU action in the coming years as is shown in Table 2.

The first thematic area highlights the participation of children as active members that have the right to be listened to and considered in decision-making. The second thematic area focuses on the importance of reducing child poverty and addressing physical as well as mental health, in combination with inclusive quality education. Combating all forms of violence against children—at home, in school, in leisure activities, in the justice system, offline and online—is the central theme of the third thematic area. The prevention and protection from violence is assured by integrated child protection systems in which services and authorities must work together. The fourth thematic area mainly concerns the actions to develop judicial proceedings appropriate to children’s needs and maturity, taking into account the rights and the best interests of the child. The Strategy also tackles the challenges of digital technologies in the fifth thematic area. It exposes the dangers around the child’s online presence, such as access to harmful content, child sexual abuse, the over exposure to screens, violation of data protection and privacy, safety as well as security. Finally, the sixth thematic area delineates the global EU actions to protect children worldwide, not only in humanitarian contexts, but also in scenarios where children are at risk of human rights violations, lack of access to education and health services, poverty and exclusion.

Annex 1 [51] aligns the Strategy’s priorities and core ideas with the Charter of Fundamental Rights of the EU [52], the United Nations Convention on the Rights of the Child [43] and the United Nations Sustainable Development Goals [53]. Annex 2 [54] specifies the most relevant EU legal and policy instruments on the rights of the child, except financial instruments.

In June 2021, the Council of the EU published the Recommendation 2021/1004 establishing a European Child Guarantee [2]. Whilst the Strategy addresses all children, the Guarantee puts focus on supporting children in need. The children in need are the persons at risk of poverty or social exclusion, under the age of 18 years. Preventing and combating social exclusion are the main goals of the Guarantee. Therefore, the Guarantee complements and is an integral part of the Strategy (Figure 2). The Member States have at their disposal Union funds to support the implementation of both the Guarantee and the Strategy.

The Guarantee has 32 pages, providing guidance and tools for Member States assuring every child in Europe at risk of poverty has access to a set of key services: (i) free early education and care; (ii) free education and school-based activities; (iii) free healthcare; (iv) at least one free healthy meal each school day; (v) adequate housing; and (vi) healthy nutrition.

The Member States must design tailored child guarantee action plans considering the national, regional as well as local needs, covering the period until 2030. The Member States should identify the specific needs of children: (i) in alternative—especially institutional—care; (ii) homeless; (iii) with disabilities; (iv) with mental health issues; (v) with a migrant background or minority ethnic origin, particularly Roma; and (vi) in precarious family situations, exposed to numerous risk factors. There is specific guidance about children in alternative care detailed below.

### Alternative Care within the EU Strategy and the Guarantee

The Strategy and the Guarantee highlight the commitment to establish child protection systems that put the child at the centre, effectively addressing the needs of children and their families. Regarding alternative care, the prevention of family separation is crucial, and poverty should never be the only argument for placing children in care. Hence, the placement of children in alternative care should consider the child’s overall situation, namely the child’s individual needs, and should ensure the respect of the rights of the child.

In order to improve the functioning of child protection systems at a national level, Member States are invited to de-institutionalise children and shift to quality family or community-based care services. In addition, to preparing children for leaving care, providing support for their independent living and social integration. Therefore, national strategies and programmes designing de-institutionalisation must be developed and integrated into a policy framework to address the social exclusion of children. Actions recommended in the implementation of this policy framework include support measures for parents or guardians, income support to families and households, stepping up investment in social protection systems, and a qualified workforce in order to provide quality services for children in need, among others.

## 4. Addressing the EU Strategy and the Guarantee into the Portuguese Alternative Care

In order to address the Strategy and the Guarantee in the Portuguese alternative care, opening the door to de-institutionalisation appears to be the course of action [37,55], supported by scientific evidence on the benefits of growing up in a family environment [10,11,12,13,56]. Quality family and community-based care, highlighted by the Strategy and the Guarantee, must be accomplished towards a progressive, but remarkable and accelerated national strategy of de-institutionalisation.

The process of change towards de-institutionalisation conducted in several countries (e.g., Greek, Georgian, Bulgarian and Armenia’s processes) and its crucial success factors may support the Portuguese implementation.

It is perceived that technical and financial support is critical in each country’s process, namely from external organisations, such as UNICEF and the EU [17,57,58], as well as political will and government leadership [57] (Greenberg and Partskhaladze, 2014). However, whenever the development of the projects is only conducted by state authorities (e.g., in the Bulgarian case), neglecting the experience of civil organizations, the process outcomes are undermined [58]. Therefore, relationships between state governments and Non-Governmental Organisations are key elements, as evidenced in the Greek, the Georgian and the Armenian processes [29,30].

Key-points are strengthening social work and the workforce, including professional training and increasing the number of personnel [9,29,30,57]; strengthening preventive and family support services; strengthening reintegration in the birth family within careful and periodical monitorization; gradually ceasing placement of children in institutions; promotion of foster care especially for younger children; and expanding small groups within residential care [17,57]. For Portugal to have significant alterations in alternative care until 2030, the present study advocates the need for integrated, simultaneous and aligned actions.

We must proclaim that residential care is referred to in literature as desirable for certain children’s needs, and the elimination of it is not argued. Residential care and family-based care must be complementary and not competitors [4]. The workforce from residential care constitutes a relevant resource for the child protection system, namely for reinforcing family support services, for a suitable kinship care service, and for a therapeutic approach in residential care. Therefore, we support qualifying and reconverting alternative care’s workforce.

Several measures in Portuguese alternative care—“supporting a child in care with another family member”; “entrusting a child to a reliable and familiar person”; special guardianship; and foster care—are considered as having the potential to be strengthened and to provide a suitable placement in the best interest of children. Hereafter, we present evidence-based recommendations to provide quality family and community-based care, as advocated by the Strategy and the Guarantee, directed to these measures and transversally the alternative care system.

### 4.1. Strengthening Kinship Care: Family Members and Reliable Persons

For those situations where the best interest of a child is to be taken care of by a family member or a person with whom he/she has established a close relationship, there are two suitable Portuguese child protection measures: “support a child in care with another family member”; and “entrust a child to a reliable and familiar person” who does not belong to the child’s family [21] (Lei no. 147/99, of 1st September in its current wording).

Relatives and reliable and familiar persons must be privileged carers for a child who needs alternative care, since they are more likely to provide family-based care and quality relationships, as recommended in the Strategy and in the Guarantee. As mentioned in the Guarantee:

“take into account the best interests of the child as well as the child’s overall situation into individual needs when placing children into institutional or foster care; ensure the transition of children from institutional or foster care to quality community-based or family-based care” [2] (p. 27).

Thus, relatives and reliable and familiar persons should be a focus of investment in Portuguese alternative care. Western Europe, the USA, New Zealand and Australia have had a shift in child protection services in the past 25 years, encouraging and increasing kinship care when children cannot live with their birth parents [59].

It is understood that kinship care placements have superior effects when compared with foster care placement [60,61,62,63]. Children in kinship care experience fewer behavioural problems, fewer mental health disorders and better well-being than do children in non-kinship care [60,61,63]. Furthermore, pre-existing bonds between a child and relative or familiar person can facilitate positive attachments; reduce the trauma children may experience when placed with strangers; reinforce a child’s sense of identity and self-esteem (family history and culture); increase the probability that children remain with their siblings; and diminish disruption in relationships and institutions (e.g., school) [59,63].

Nevertheless, there are several concerns regarding: (i) ability of kinship carers to protect children from neglect and abuse by birth parents; (ii) quality of their care; and (iii) outcomes for the child [59]. Hence, kinship carers may be less prepared for childcare responsibilities [63].

In Portugal, since 2008, in foster care (detailed below), carers may not have blood ties with the foster child [64], justified by the fact that there were already two measures for placements within a family or familiar person. However, these two measures differ from foster care in terms of support and benefits from the social services. For example, in the measure “supporting a child in care with another family member”, support consists of psycho-pedagogical and social assistance, and if in need a financial benefit may be activated [65]. Hence, mandatory training for carers is not to be provided.

Disparities between foster care and kinship care in terms of support are observed in other countries too [62,66]. Therefore, as recommendations for policy and practice, research suggests providing more assistance services, including financial assistance, support services and training to kinship carers [62], including respite childcare; guidance from child welfare services (e.g., clear visitation guidelines) and more active involvement of child protection services social workers [63]. It is intended that a closer collaboration between services could improve kinship carers’ access to resources to meet the child’s needs [66].

Riehl and Shuman [63], within the best and inspiring practices on how other countries support kinship families, highlight a “Kinship Care Portal” (e.g., in Georgia) that provides kinship carers with access to information, referrals, and resources. In addition, pointing out specialising kinship care workers and adopting a “family support model” to attend the triad: child, birth parent, and reliable member’s specific needs (e.g., Australian agencies).

Therefore, this study advocates more support to carers who are a member of the family (e.g., grandparents; uncles) or a reliable person (e.g., parents’ friends; neighbours). A better alignment and the same support between kinship care and foster care must be provided. The arguments include that placements have similar challenges and expenses, and the differences between measures (e.g., training and licencing requirements) may influence a child’s mental health outcomes [62].

With a view to transition towards family and community-based care in Portugal, this study recommends an alignment with foster care for kinship care: (i) financial support to tackle the child needs, including extracurricular and cultural activities to prevent social exclusion; (ii) preparation prior to the placement, and training for the relative/s or the reliable person/s; (iii) ongoing support from child protection system professionals; and (iv) creation of specialised and qualified teams to support kinship carers and the child within their specific needs.

This study suggests that whenever a child needs permanent alternative care, a placement with a relative or a reliable person may be the best for the child, and the measure may become a special guardianship.

### 4.2. Room for Special Guardianship

Portuguese special guardianship has no exact equivalent in other judicial systems, having some similarities with England’s special guardianship [28]. Therefore, the research evidence on special guardianship presented here is limited to studies from these two countries.

Despite the evident benefits of special guardianship—a family-based care alternative to child long term institionalisation, a flexible measure that allows relatives and non relatives to be special guardians and, whenever possible, the preservation of the relationship with the birth parents—its use in the Portuguese context is minimal [67,68]. As already stated, in 2020, only two special guardianships were made effective [31]. Moreover, special guardianship was first introduced to promote de-institutionalisation [28]. What can be done to make room for the special guardianship as an effective option for children in need?

Dias [69] and Ferreira [68] advocate the importance of financial support to special guardians. Although special guardians already have social benefits and rights similar to birth parents, financial support must be provided to tackle the child’s physical and mental health, and educational needs, including the participation in sport, leisure or cultural activities. The Guarantee states that “[…] to provide for effective access or effective and free access to key services, Member States should, […] provide such services or provide adequate benefits so that parents or guardians of children in need are in a position to cover the costs or charges of those services” [2] (p. 7).

In order to be aligned with foster care, special guardians should receive proper training and ongoing support. In a focus group study conducted in England with a total of 44 family justice practitioners, including lawyers, social workers and guardians, one of the conclusions suggests the need for a robust system of preparation and training for prospective special guardians [70,71]. Furthermore, one of the key messages from special guardianship research in England is the need of ongoing support for special guardians, not only to deal with emotional and behavioural difficulties of the children, but along with the occasional problematic arrangements with birth parents [70]. Skilled professionals from residential or foster care can be important resources for the support work with special guardians. Another important finding from special guardianship research in England concerns children’s stability: special guardianship is a stable option having a very low rate of return, but the risk of placement disruption increases for children placed with unrelated carers [70,71].

A view to transition towards family and community-based care in Portugal, this study recommends special guardianship: (i) financial support to tackle the child needs, including extracurricular and cultural activities to prevent social exclusion; (ii) preparation and specialised training for prospective special guardians; (iii) ongoing support from child protection professionals, especially for placements involving children with unrelated carers; (iv) recruitment of special guardians; and (v) a definition of goals and action plans related to special guardianship in the Portuguese Strategy.

### 4.3. Foster Care—What Is Still to Be Done?

Since 2015, the Portuguese law [23] states clearly the relevance of foster care instead of residential care placements, namely for children up to 6 years old. In 2019 and 2020 foster families support was reinforced in terms of social, financial, fiscal, and labour rights [35,36,72]. For example, financial support shifted from around EUR 330 to a minimum of EUR 522 monthly, depending on a few conditions.

Despite progress, facts and figures do not coincide with law improvements [34]. Therefore, moving from residential care to foster care must be urgent and it should be an evidence-based process.

The Guidelines for the Alternative Care of Children [7] highlight the need for appropriate foster carer’s preparation and training, and emphasising the need for a matching procedure in order to meet a child’s needs and to maximise a positive outcome for the placement. Moreover, and considering foster carers’ lack of opportunities for expressing their concerns and ideas that could positively influence policy on this alternative care option [6,7], they should be encouraged to form an association in Portugal, to provide mutual support through a forum where they can express concerns and gain from the experience of others, and to obtain their views to influence practice and policy [6,7]. Additionally, trained foster carers can also be a role model of sensitive and positive parental care to the birth parents leading to the rehabilitation of the family. It may occur during contact visits (between the birth parent and child), made to the foster family home or on a pre-arranged appointment at a day care facility [73].

Cantwell and colleagues [6] point out substantial tactics, and we intend evidence listed above to be read as implications for policymaking:

1. Investing resources so foster care is available widely;
2. Providing adequate financing;
3. Regulating and monitoring;
4. Providing flexible placements to address children’s needs (e.g., emergency placements, respite care, short term and longer-term placements);
5. Ensuring children and foster carers participation and mechanisms for complaints; requiring that siblings are placed together;
6. Guaranteeing children have contact with their parents, wider family, friends and community;
7. Providing appropriate support and training for carers, especially for those who care for children with disabilities and other special needs, and including topics on child development and attachment, children’s rights and child well-being;
8. Ensuring day care and respite care, health and education services whenever needed.

For developing a foster care system, it is central to increase the number of foster families towards both strategies, recruiting new families as well as retaining the experienced families [37]. A systematic review [74] on recruitment and retention found that the intention to become a foster parent is largely influenced by motivational factors; personal and family characteristics; individual values and beliefs; social context influences; and perceived familiarity with the child protection system. Therefore, it suggests that recruitment campaigns should emphasize the intrinsic motivational factors and the resources needed to provide quality; appeal to moral responsibility, as well as to the difference that individuals could make in children’s lives; and disseminate accurate information (considering adequate knowledge about foster care is relevant in decisions to foster).

These authors [74] analysing several studies, found that retention of foster families is closely related to factors within the child protection system, such as foster child characteristics; personal or family characteristics; and placement challenges, as well as relationship with services and professional support, in line with Portuguese studies, e.g., Diogo and Branco [75]. It is highlighted that not only close and warm relationships between professionals and foster families are relevant to helping them adequately deal with diverse challenges, but also specific training is needed—on empathic relationships to prevent significant problems between foster families and services, and on educational strategies, expectations of the foster child and Foster Care system, and promoting positive attitudes towards the foster child’s family and their life history [74].

Miller and colleagues [76] identified carer (personal and family) factors associated with foster–placement success and breakdown, including higher cognitive empathy of the carer; a high level of social support from the family; a high-quality carer–partner relationship; higher levels of caregiving and role–carer demand satisfaction, and a good match; fewer conflicts and better relationship between the carer and foster child. Implications for practice include additional evaluations during screening processes with a focus on these key markers, more emphasis on developing support networks amongst carers’ friends and family [37] and greater involvement of carer partners in screening and training processes [76].

Matching a child with foster carers may be instrumental in determining outcomes for a child, and sustainability of a placement [77,78]. Matching is a complex decision-making process, and a range of factors (e.g., siblings, ethnicity/culture, behaviour, carer experience and expectations, parenting style or organizational factors) are considered [78,79] during the process of selecting a foster family who is the best fit for a child. Participation of children, birth parents and foster carers in the matching decision-making process has the potential to improve the outcomes of a placement, empowers stakeholders and can diminish negative effects. However, professionals should be aware that assumptions, timing and feasibility can restrict the influence of these stakeholders [80].

To transition towards family and community-based care in Portugal, this study recommends for foster care: (i) evidence-based recruitment campaigns to increase the quality and diversity of carers instead of just increasing the number of foster carers; (ii) foster care services for children in the whole country preventing inequality; (iii) financial support to tackle the child’s needs, including extracurricular and cultural activities to prevent social exclusion; (iv) flexible placements to address children’s needs (e.g., just during weekends, respite care); (v) value foster carers according to their experience (e.g., be a role model for other carers); and (vi) empower carers and provide mutual support toward the creation of a foster families’ association.

### 4.4. Global Dimensions of Alternative Care

Several and detailed recommendations on three services—kinship care, special guardianship, and foster care—have been stated. Alternative care has transversal aspects, thus recommendations regarding global dimensions of it are presented in this last topic. Nevertheless, shifting towards de-institutionalisation, this paper argues that consultancy and technical support of external and experienced organizations, such as UNICEF, seem to be relevant for Portugal as has been the case in other countries.

To transition towards family and community-based care in Portugal, this study recommends for alternative care: (i) review the nature of the measures, particularly support and terminology (aligning with international concepts for an easier analysis and comparison); (ii) create suitable indicators on alternative care and monitoring it continuously; (iii) gathering and treatment of data from the diverse services (Child Protection Commissions and courts) in an integrated mode and publishing periodically (quarterly is required); qualitative and quantitative data must be equally privileged; (iv) promote scientific studies including all stakeholders from the child protection system, in order to generate evidence-based policy and practice decisions; and (iv) create a family-based care culture for the Portuguese society and child protection system workforce, seeing beyond foster care.

## 5. Conclusions

This study generates knowledge on evidence-based policy and practice to implement the Strategy and the Guarantee concerning alternative care in Portugal.

On the one hand, the existing services involving family and community-based care should be prioritised and strengthened. Recommendations held in common include specific and continuous support for children and carers, such as previous preparation and training as well as suitable financial support to ensure that a child does not grow up at risk of social exclusion. On the other hand, the alternative care system must improve. The recommendations highlight the creation of suitable indicators on alternative care to gather an integrated national picture of children in care. Priority should be given to quantitative and qualitative data together with regular monitoring and minimum quarterly publication. The measures concerning family based-care should be revised in order to cover more financial and social support. The revision should also include an alignment in terminology with the international concepts.

Limitations of the study are based on the lack of systematic literature reviews on this topic and of Portuguese data on kinship care. Finally, research is needed to evaluate the Portuguese and other Member State’s implementation of the Strategy and the Guarantee in order to generate an evidence-based policy.

## Figures and Tables

**Table 1:** A proposal categorisation of alternative care concepts. Source: own elaboration.

	Alternative Care Concepts			
	Family-Based Care			Residential Care
	Kinship Care	Foster Care	Other	
Portuguese child protection and civil measures on alternative care	Support the child in care with other family member	Foster care	Special guardianship	Residential care
	Entrust the child to a reliable and family person			

**Table 2:** The EU Strategy on the Rights of the Child’s thematic areas and the European Child Guarantee’s target groups and key services. Source: own elaboration.

EU Stratrgy on the Rights of the Child ALL Children					
Thematic areas					
Participation in political and democratic life: An EU that empowers children to be active citizens and members of democratic societies	Socioeconomic inclusion, health and education: An EU that fights child poverty, promotes inclusive and child-friendly societies, health and education systems	Combating violence against children and ensuring child protection: An EU that helps children grow free from violence	Child-friendly justice: An EU where the justice system upholds the rights and needs of children	Digital and information society: An EU where children can safely navigate the digital environment and harness its opportunities	The Global Dimension: An EU that supports, protects and empowers children globally, including during crisis and conflcit
European Child Guarantee					
Children in need, namely:			Access to key kervices:		
In alternative, especially instititional care	Homeless children	With disabilities	Free early education and care, education and school-based activities, healthcare and at least one healthy meal each school day		
With mental health issues	With a migrant background or minority ethic origin (particularly Roma)	In precarious family situations	Adequate housing and healthy nutrition		

## Appendix A

**Table A1:** Portuguese National Strategy for the Rights of the Child’s Framework.

Priority	Strategic Aim	Support References
Priority I promote wellbeing and equal opportunities	SA1 Ensure a standard of living suitable to the development of children, through an efficient allocation of social and fiscal support	Convention on the Rights of the Child (CRC) 26.1; CRC 27; Recommendation (REC) 16; REC 58
	SA2 Promote a safety and healthy environment	CRC 28; REC 30
	SA3 Invest in prevention and promote physical and mental health support in childhood, in order to develop healthy generations	CRC 24; CRC 25; REC 50; CRC 19.2; REC 52; CRC 33; REC 54; CRC 28
	SA4 Ensure children access to quality playful, entertaining and sporting activities	CRC 31; CRC 29; REC 26
	SA5 Ensure access to quality inclusive education to all children, contributing for their physical, cognitive, social and emotional development	CRC 28; REC 60 (b); CRC 29; REC 60 (c); REC 60 (d); CRC 26.2; CRC 27
	SA6 Qualify and reinforce measures, programmes, services and social responses, as well as support for disabled children and their families	CRC 23; REC 46
	SA7 Support migrants children integration, including refugees and asylum seekers, descent of migrants and Roma	CRC 2; CRC 8; CRC 29; CRC 30 REC 26
Priority II support families and parenthood	SA8 Foment competences for a positive parenthood and shared parenthood responsability	CRC 18.2; REC 40
	SA9 Qualify measures, programmes, and social and health responses for children within an integrated family approach	CRC 26; CRC 18.3; REC 16; CRC 24; REC 56; CRC 6; CRC 27; CRC 20; REC 42; CRC 21; REC 44; CRC 25; REC 42 (d)
Priority III promote access to information and to participation for children	SA10 Promote information and training on the rights of children	CRC 42; REC 22; Lanzarote Convention (LC) 9; CRC 28; CRC 29; REC 30 (a); CRC 2; CRC 4; CRC 3.1; CRC 19
	SA11 Promote participation and exercise of citizenship of children	CRC 2; CRC 8; CRC 13; CRC 14; CRC 29; CRC 42; REC 26 (a); REC 26 (b); REC 60 (e); CRC 7; CRC 12; REC 32 (a); REC 32 (b); REC 32 (c); CRC 12; REC 32; LC 9; LC 35; CRC 4; CRC 12; CRC 40; REC 10; REC 32 (a); REC 66; CRC 15; CRC 31
Priority IV prevent and combat violence against children	SA12 Prevent and act on different forms of violence against children, promoting a non-violence culture	CRC 34; CRC 35; CRC 36; CRC 39 REC 24; REC 26 (b); REC 36; LC 4, 5, 6, 7 e 8; Optional Protocol on Sales of Children (OP -SC); CRC 19; CRC 29; REC 60 (e); REC 46; CRC 28.2; CRC 17; CRC 4
	SA13 Promote knowleadge on different forms of violence against children and qualify responses	CRC 19; REC 28; REC 46; CRC 39; CRC 42; REC 26 (b); LC5C; REC 24
Priority V promote instruments and scientific knowledge production for a global view on the rights of children	SA14 Tailor national legislation on children to the CRC	CRC 34; CRC 40; REC 10; REC 36 OP-SC; LC 31
	SA15 Concept and implement a data gathering and analysis system on children	CRC 4; CRC 17; REC 18; LC 10.2

Source: own elaboration base on Resolução do Conselho de Ministros no. 112/2020, of 18 December [18].

## References

[1] European Commission (2021). Communication from the Commission to the European Parliament, the Council, the European Economic and Social Committee and the Committee of the Regions EU strategy on the Rights of the Child (COM/2021/142 final). European Union, European Commission. https://eur-lex.europa.eu/legal-content/en/TXT/?uri=CELEX%3A52021DC0142.

[2] Council Recommendation 2021/1004 Establishing a European Child Guarantee. European Union, Council of the European Union. http://data.europa.eu/eli/reco/2021/1004/oj.

[3] Dolan K. (2020). Group Homes in the Foster Care System: A Literature Review. Locus Seton Hall J. Undergrad. Res..

[4] Carvalho M.J., Diogo E., Magalhães E., Baptista J. (2021). Acolhimento Familiar de Crianças e Jovens: O nó cego da proteção à infância em Portugal. Acolhimento Familiar de Crianças e Jovens em Perigo—Manual Para Profissionais.

[5] Bergström M., Cederblad M., Håkansson K., Jonsson A.K., Munthe C., Vinnerljung B., Wirtberg I., Östlund P., Sundell K. (2019). Interventions in Foster Family Care: A Systematic Review. Res. Soc. Work. Pract..

[6] Cantwell N., Davidson J., Elsley S., Milligan I., Quinn N. (2012). Moving Forward: Implementing the ‘Guidelines for the Alternative Care of Children’.

[7] United Nations General Assembly (2009). Guidelines for the Alternative Care of Children. Resolution A/RES/64/142. http://www.un.org/ga/search/view_doc.asp?symbol%20=%20A/RES/64/142.

[8] European Social Network (2011). Developing Community Care. European Social Network. https://www.esn-eu.org/sites/default/files/publications/2011_Developing_Community_Care_Report_EN.pdf.

[9] Gilligan R. “The De-institutionalization Process in Ireland”—What have we learned?. Proceedings of the De-Institutionalization of Childcare: Investing in Change.

[10] Negrão M., Moreira M., Veríssimo L., Veiga E. (2019). Conhecimentos e perceções públicas acerca do acolhimento familiar: Contributos para o desenvolvimento da medida. Anal. Psicol..

[11] Freitas S. (2019). Why Do People Become Foster Parents? From the Literature to Empirical Evidence. Master’s Thesis.

[12] Gonçalves M. (2017). Crescer em Famílias de Acolhimento: Histórias de Vida de Jovens—Adultos. Master’s Thesis.

[13] Schofield G., Beek M., Sargent K., Thoburn J. (2000). Growing Up in Foster Care.

[14] McCall R., Groark C., Rygaard N. (2014). Global Research, Practice, and Policy Issues on the Care of Infants and Young Children at Risk: The Articles in Context. Infant Ment. Health J..

[15] Soares I., Belsky J., Oliveira P., Silva J., Marques S., Baptista J., Martins C. (2014). Does early family risk and current quality of care predict indiscriminate social behavior in institutionalized Portuguese children?. Attach. Hum. Dev..

[16] Davidson J.C., Milligan I., Quinn N., Cantwell N., Elsley S. (2016). Developing family-based care: Complexities in implementing the UN Guidelines for the Alternative Care of Children. Eur. J. Soc. Work.

[17] Terziev V., Arabska E. (2016). Process of Deinstitutionalization of Children at Risk in Bulgaria. Procedia Soc. Behav. Sci..

[18] Resolução do Conselho de Ministros no. 112/2020 *Diário da República*, 18 December 2020, 1.ª série, 245. https://files.dre.pt/1s/2020/12/24500/0000200022.pdf.

[19] (1991). Decreto-Lei no. 189/91, of 17th May, *Diário da República*, 113, Série I-A. https://files.dre.pt/1s/1991/05/113a00/26352640.pdf.

[20] Gersão E., Sacur B.M., Francisco R., Pinto H.R. (2021). Promoção de direitos e proteção de crianças e jovens: Passado, presente e cami-nhos de futuro [Promotion of rights and protection of children and youth: Past, present and future paths]. Atores e Dinâmicas no Sistema de Promoção e Proteção de Crianças e Jovens.

[21] (1999). Lei no. 147/99, of 1st September, *Diário da República*, 204, Série I-A. http://www.pgdlisboa.pt/leis/lei_mostra_articulado.php?nid=545&tabela=leis.

[22] (2003). Lei no. 31/2003, of 22nd of August, *Diário da República*, 193, Série I-A. http://www.pgdlisboa.pt/leis/lei_mostra_articulado.php?nid=546&tabela=leis.

[23] (2015). Lei no. 142/2015, of 8th September, *Diário da República,* 175, Série I. https://www.seg-social.pt/documents/10152/9597919/Lei_n_142_2015_09_08/7059a660-0ddc-4547-9a99-ded7aec210ad.

[24] Lei no. (2017). Lei no. 23/2017, of 23th of May, *Diário da República*, 99, Série I. https://files.dre.pt/1s/2017/05/09900/0249402494.pdf.

[25] Lei no. (2018). Lei no. 26/2018, of 5th July, *Diário da República*, 128, 1.ª série. https://files.dre.pt/1s/2018/07/12800/0290202903.pdf.

[26] (2009). Lei no. 103/2009, of 11th September, *Diário da República*, 177, Série I. https://dre.pt/dre/legislacao-consolidada/lei/2009-34513875.

[27] (2010). Decreto-Lei no. 121/2010, of 27th October, *Diário da República*, 209, Série I. https://www.pgdlisboa.pt/leis/lei_mostra_articulado.php?nid=1287&tabela=leis&so_miolo=.

[28] Oliveira G. (2019). Adoção e Apadrinhamento Civil [Adoption and Special Guardianship].

[29] Brodie I., Pearce J. (2017). Violence and Alternative Care: A Rapid Review of the Evidence. Psychol. Health Med..

[30] UNICEF Europe and Central Asia Regional Office (2021). The European Child Guarantee Phase III of the Preparatory Action: “Testing the EU Child Guarantee in the EU Member States”. https://www.unicef.org/eca/european-child-guarantee.

[31] CNPDPCJ—Comissão Nacional de Promoção dos Direitos e Proteção das Crianças e Jovens (2021). Relatório Anual de Avaliação da Atividade das CPCJ 2020. https://www.cnpdpcj.gov.pt/documents/10182/16406/Relat%C3%B3rio+Anual+da+Atividade+das+CPCJ+do+ano+2020/2a522cda-e8ba-40fe-9389-47fa5966f7ed.

[32] Pordata. https://www.pordata.pt/Portugal/Popula%c3%a7%c3%a3o+residente++m%c3%a9dia+anual+total+e+por+grupo+et%c3%a1rio-10-1141.

[33] DGPJ-Direção Geral de Política de Justiça (2021). Movimento de Processos de Promoção e Proteção nos Tribunais Judiciais de 1.ª Instância, nos anos de 2011 ao 1º Trimestre de 2021.

[34] ISS—Instituto da Segurança Social, Instituto Público (2021). CASA, 2020—Relatório de Caracterização Anual da Situação de Acolhimento das Crianças e Jovens. https://www.seg-social.pt/documents/10152/13200/CASA+2020.pdf/b7f02f58-2569-4165-a5ab-bed9efdb2653.

[35] (2019). Decreto-lei no. 139/2019, 16th of September, *Diário da República*, 177, Série I. http://www.pgdlisboa.pt/leis/lei_mostra_articulado.php?artigo_id=3204A0035&nid=3204&tabela=leis&pagina=1&ficha=1&so_miolo=&nversao=.

[36] (2020). Portaria no. 278-A/2020, 4th of December, *Diário da República*, 236, Série I. https://dre.pt/dre/detalhe/portaria/278-a-2020-150343971.

[37] Diogo E. (2018). Ser Família de Acolhimento em Portugal, Motivações e Experiências [Being a Foster Family in Portugal—Motivations and Experiences].

[38] Department for Education (2019). Children Looked after in England (Including Adoption), Year Ending 31 March 2019. https://www.gov.uk/government/statistics/children-looked-after-in-england-including-adoption-2018-to-2019.

[39] Furey E., Canavan J. (2019). A Review on the Availability and Comparability of Statistics on Child Protection Welfare, Including Children in Care Collated by Tusia: Child and Family Agency with Statistics Published in Other Jurisdictions.

[40] Ministerio de Derechos Sociales y Agenda 2030 (2020). Boletín de Datos Estadísticos de Medidas de Protección a la Infancia. Boletín número 22, Datos 2019. https://observatoriodelainfancia.vpsocial.gob.es/productos/pdf/BOLETIN_22_final.pdf.

[41] United Nations, Committee on the Rights of the Child Concluding Observations on the Combined Third and Fourth Periodic Report of Portugal. CRC/C/PRT/CO/3-4. https://tbinternet.ohchr.org/Treaties/CRC/Shared%20Documents/PRT/CRC_C_PRT_CO_3-4_16303_E.pdf.

[42] United Nations, Committee on the Rights of the Child (2019). Concluding Observations on the Combined Fifth and Sixth Periodic Report of Portugal. CRC/C/PRT/CO/5-6. https://www.provedor-jus.pt/documentos/Observacoes_Finais_Comite_Dtos_Crianca.pdf.

[43] United Nations (1989). Convention on the Rights of the Child. http://www.ohchr.org/EN/ProfessionalInterest/Pages/CRC.aspx.

[44] Flick U. (2005). Métodos Qualitativos na Investigação Científica.

[45] Bryman A. (2012). Social Research Methods.

[46] Optional Protocol to the Convention on the Rights of the Child on the Involvement of Children in Armed Conflict. https://www.ohchr.org/EN/ProfessionalInterest/Pages/OPACCRC.aspx.

[47] (2000). Optional Protocol to the Convention on the Rights of the Child on the Sale of Children, Child Prostitution and Child Pornography. https://www.ohchr.org/EN/ProfessionalInterest/Pages/OPSCCRC.aspx.

[48] (2012). Optional Protocol to the Convention on the Rights of the Child on a Communications Procedure. https://www2.ohchr.org/english/bodies/crc/docs/CRC-OP-IC-ENG.pdf.

[49] (2006). United Nations Convention on the Rights of Persons with Disabilities. https://www.ohchr.org/en/hrbodies/crpd/pages/conventionrightspersonswithdisabilities.aspx.

[50] Council of Europe (2016). Council of Europe Strategy for the Rights of the Child (2016–2021). https://rm.coe.int/CoERMPublicCommonSearchServices/DisplayDCTMContent?documentId=090000168066cff8.

[51] European Commission (2021). Annex to the EU Strategy on the Rights of the Child. EU and International Frameworks. European Union, European Commission. https://ec.europa.eu/info/sites/default/files/childrights_annex1_2021_4_digital_0.pdf.

[52] European Union (2010). Charter of Fundamental Rights of the European Union. Off. J. Eur. Union.

[53] United Nations General Assembly (2015). Transforming Our World: The 2030 Agenda for Sustainable Development. Resolution A/RES/70/1. https://www.un.org/ga/search/view_doc.asp?symbol=A/RES/70/1andLang=E.

[54] European Commission (2021). Annex to the EU Strategy on the Rights of the Child. EU Acquis and Policy Documents on the Rights of the Child. European Union, European Commission. https://ec.europa.eu/info/sites/default/files/childrights_annex2_2021_4_digital_0.pdf.

[55] Chaves S. (2018). Constrangimentos e Potencialidades Associados à Medida de Acolhimento Familiar de Crianças e Jovens. Master’s Thesis.

[56] Li D., Chng G., Chu C. (2019). Comparing Long-Term Placement Outcomes of Residential and Family Foster Care: A Meta-Analysis. Trauma Violence Abus..

[57] Greenberg A., Partskhaladze N. (2014). How the Republic of Georgia Has Nearly Eliminated the Use of Institutional Care for Children. Infant Ment. Health J..

[58] Ivanova V., Bogdanov G. (2013). The Deinstitutionalization of Children in Bulgaria—The Role of the EU. Social Policy and Administration.

[59] Skoglund J., Thørnblad R. (2019). Kinship Care or Upbringing by Relatives? The Need for ‘New’ Understandings in Research. Eur. J. Soc. Work.

[60] Winokur M., Holtan A., Batchelder K. (2014). Kinship Care for the Safety, Permanency, and Well-Being of Children Removed from the Home for Maltreatment: A Systematic Review. Retrieved from Cochrane Database of Systematic Reviews. https://www.cochranelibrary.com/cdsr/doi/10.1002/14651858.CD006546.pub3/epdf/abstract.

[61] Bergthold A. The Effect of Kinship Foster Care Compared to Non-Kinship Foster Care on Resiliency. Leadership Connection 2018. https://digitalcommons.cedarville.edu/cgi/viewcontent.cgi?article=1455&context=research_scholarship_symposium.

[62] Xu Y., Bright L. (2018). Children’s Mental Health and Its Predictors in Kinship and Non-Kinship Foster Care: A Systematic Review. Child. Youth Serv. Rev..

[63] Riehl C., Shuman T. (2020). Children Placed in Kinship Care: Recommended Policy Changes to Provide Adequate Support for Kinship Families. Child. Leg. Rights J..

[64] (2008). Decreto-Lei no. 11/2008, of 17th January, *Diário da República*, Série I. https://dre.pt/dre/legislacao-consolidada/decreto-lei/2008-34455775-53087975..

[65] (2008). Decreto-Lei no. 12/2008, of 17th January, *Diário da República*, Série I. https://dre.pt/dre/legislacao-consolidada/decreto-lei/2008-34455875..

[66] Xu Y., Bright L., Ahn H., Huang H., Shaw T. (2020). A New Kinship Typology and Factors Associated with Receiving Financial Assistance in Kinship Care. Child. Youth Serv. Rev..

[67] Alfaiate A.R., Ribeiro G. (2013). Reflexões a Propósito do Apadrinhamento Civil [Reflections on Special Guardianship]. Rev. Do Cent. De Estud. Judiciários.

[68] Ferreira E. (2019). O apadrinhamento civil como alternativa ao acolhimento permanente de crianças e jovens [Special Guardianship as an alternative to permanent care of children and youth]. Configurações.

[69] Dias C., Gonçalves L.C. (2012). Algumas notas em torno do Regime Jurídico do Apadrinhamento Civil [Some notes about special guar-dianship legal regime]. Estudos em Homenagem ao Professor Doutor Heinrich Ewald Hörster.

[70] Simmonds J., Harwin J., Brown R., Broadhurst K. (2019). Special Guardianship: A Review of the Evidence. Summary Report. Nuffield Family Justice Observatory. https://www.nuffieldfjo.org.uk/wp-content/uploads/2021/05/NuffieldFJO-Special-Guardianship-190731-WEB-final.pdf.

[71] Simmonds J., Harwin J. (2020). Making Special Guardianship Work for Children and Their Guardians. Briefing paper. Nuffield Family Justice Observatory. https://www.nuffieldfjo.org.uk/wp-content/uploads/2021/05/making_special_guardianship_work_briefing_paper.pdf.

[72] (2019). Lei no. 47/2019, of 8th July. Diário da República, 1.ª série, 128. https://www.seg-social.pt/documents/10152/16485882/Lei_47_2019.pdf/b0e3416d-6ef6-4080-873f-de81f1e5b21d..

[73] European Commission Daphne Programme—Directorate-General Justice and Home Affairs (2007). De-Institutionalising and Transforming Children’s Services—A Guide to Good Practice. University of Birmingham. https://www.globaldisabilityrightsnow.org/sites/default/files/related-files/262/De-institutionalizing%20and%20Transforming%20Children%E2%80%99s%20Services%20A%20Guide%20to%20Good%20Practice.pdf.

[74] Gouveia L., Magalhães E., Pinto V. (2021). Foster Families: A Systematic Review of Intention and Retention Factors. J. Child. Fam. Stud..

[75] Diogo E., Branco F. (2020). The Foster Family Process to Maintain the Will to Remain in Foster Care—Implications for a Sustainable Programme. Sustainability.

[76] Miller L., Randle M., Dolnicar S. (2019). Carer Factors Associated with Foster-Placement Success and Breakdown. Br. J. Soc. Work.

[77] Diogo E., Branco F. (2017). Being a Foster Family in Portugal—Motivations and Experiences. Societies.

[78] Haysoma Z., McKibbinb G., Shlonskyc A., Hamiltond B. (2020). Changing Considerations of Matching Foster Carers and Children: A Scoping Review of the Research and Evidence. Child. Youth Serv. Rev..

[79] Zeijlmans K., López M., Grietens H., Knorth E. (2017). Matching Children with Foster Carers: A Literature Review. Child. Youth Serv. Rev..

[80] Zeijlmans K., López M., Grietens H., Knorth E. (2019). Participation of Children, Birth Parents and Foster Carers in the Matching Decision. Paternalism or Partnership?. Child. Abus. Rev..

